# Tele-Psychiatry Assessment of Post-traumatic Stress Symptoms in 100 Patients With Bipolar Disorder During the COVID-19 Pandemic Social-Distancing Measures in Italy

**DOI:** 10.3389/fpsyt.2020.580736

**Published:** 2020-12-03

**Authors:** Claudia Carmassi, Carlo Antonio Bertelloni, Valerio Dell'Oste, Filippo Maria Barberi, Alessandra Maglio, Beatrice Buccianelli, Annalisa Cordone, Liliana Dell'Osso

**Affiliations:** Department of Clinical and Experimental Medicine, University of Pisa, Pisa, Italy

**Keywords:** tele-psychiatry, post-traumatic stress symptoms, COVID-19, bipolar disorder, mood disorder, pandemic

## Abstract

The acute phase of the COrona VIrus Disease-19 (COVID-19) emergency determined relevant stressful burdens in psychiatric patients, particularly those with chronic mental disorders such as bipolar disorder (BD), not only for the threat of being infected but also for the strict lock-down and social-distancing measures adopted, the economic uncertainty, and the limited possibilities to access psychiatric services. In this regard, telepsychiatry services represented a new important instrument that clinicians could adopt to monitor and support their patients. The aim of the present study was to investigate acute post-traumatic stress symptoms (PTSS) reported by patients with BD followed in the framework of a telepsychiatry service, set up in the acute phase of the COVID-19 outbreak at the psychiatric clinic of the University of Pisa (Italy). A sample of 100 patients were consecutively enrolled and assessed by the IES-r, GAD-7, HAM-D, and YMRS. Patients reported a mean (±SD) IES-r total score of 18.15 ± 13.67. Further, 17% of the sample reported PTSS (IES-r > 32), 17% depressive symptoms (HAM-D > 17), and 26% anxiety symptoms (GAD-7 > 10). Work and financial difficulties related to the COVID-19 pandemic and anxiety symptoms appeared to be positively associated with the development of acute PTSS. Acute manic symptoms appeared to be protective. The data of the present study suggest the relevance of monitoring patients with BD exposed to the burden related to the COVID-19 outbreak for prompt assessment and treatment of PTSS.

## Introduction

Increasing literature suggests how COVID-19 and the related quarantine or social-distancing measures, adopted in the acute phase, may have represented a traumatic experience that could have affected mental health and well-being of exposed individuals (1, 2). At the end of April 2020, after 55 days of national lock-down during the so-called *first phase* of the pandemic, in Italy the number of COVID-19 cases exceeded 200,000 units and the death count 31,000 units. During this period, most of the population lived in home-confinement environments avoiding social interactions, and the COVID-19 outbreak may have represented a relevant trauma not only for the risk of being infected but also for the strict lock-down and social-distancing measures, the economic uncertainty, and the limited possibilities to access mental health services. The first studies investigating mental health stress burden on a general population exposed to the COVID-19 pandemic showed high levels of depression, anxiety, and post-traumatic stress symptoms (PTSS) (3–7). Particularly, Liu et al. (5) reported a 7% prevalence rate of clinically significant PTSS in the Chinese general population living in the hardest-hit areas during the COVID-19 epidemic. Another recent study showed post-traumatic stress disorder (PTSD) and depression rates of 2.7 and 9.0%, respectively, among 2,485 home-quarantined Chinese University students (6).

Despite the fact that psychological burden of the COVID-19 emergency has been very different across different countries and strongly related to specific regional conditions (5, 8), it seems very likely that the pandemic will affect long-term mental health in populations with low degree of resilience, such as patients with chronic psychiatric disorders such as bipolar disorder (BD). Comorbidity between BD and PTSD has been widely investigated in the literature, showing prevalence rates ranging between 4 and 40% for PTSS, and between 9 and 20% for PTSD diagnosis (9, 10).

As the general population, subjects affected by pre-existing mental disorders faced several stressors during the period of national lockdown in Italy, such as isolation, loneliness, sudden bereavement without being able to bury their loved ones, and fear of ultimately and suddenly losing their own lives (11). Despite the fact that some authors did not find any detrimental effect of the COVID-19 outbreak on the social inclusion and well-being in subjects with mental health problems (12), pandemic concerns might have represented a significant stressor, particularly for subjects with severe mental illness, which were vulnerable to the risk of anxiety and mood relapse (13). Further, social distancing limited access to treatment and support centers, including mental health services, day programs, and congregate care settings, as well as limiting contact with families and loved ones, enduring increasingly prohibitive visitor policies (13–16). On one hand, some individuals with severe mental disorders might have been less impacted by the public health restrictions, because they lived already “socially distanced,” with minimal interpersonal contacts outside of their immediate living environment and necessities, whether as a result of their symptoms, societal marginalization, or personal choice; on the other hand, in most of these patients the lockdown measures further reduced and collapsed the weak existing social networks (17). Consistently, Hao et al. (11) reported more severe acute PTSS, anxiety, and depression symptoms among Chinese psychiatric patients during the peak of the COVID-19 epidemic with strict lockdown measures than in healthy controls, with more than one-quarter of patients reporting PTSS.

A major issue for clinicians and researchers in psychiatry is now to detect which impact the COVID-19 pandemic will have on psychiatric long-term care going forward. The sudden changes occurred could have significantly impacted psychiatric patients' mental health as well as having reduced their opportunity to access psychiatric services (13, 16). Because of the pandemic, psychiatric inpatients were exposed to a high risk of COVID-19 infection. Consequently, most of the psychiatric patients in maintenance phase or with minor symptoms received their treatment at home to reduce the risk of infection, and clinicians first adopted telepsychiatry services to monitor their patients (13, 18, 19). Further, a significant portion of psychiatric patients that were quarantined in their homes resulted in social isolation and loneliness, which could fuel anxiety and mood destabilization secondary to physical distancing and shelter-in-place guidelines. Therefore, connecting with these individuals seemed essential to provide treatment for acute psychiatric concerns as well as to continue treatment of chronic illness. Nowadays, a rich literature supports telepsychiatry, demonstrating excellent acceptance and non-inferior outcomes across ages, conditions, cultures, and languages (20). Remote consultation via telemedicine to overcome the rules of social distancing during the peak of the COVID-19 pandemic had been rapidly embraced in several countries (8, 21–23) and operational instructions for mental health departments and community hospitals, such as tele-health and phone check-ins, had been also promptly set up in some hospitals in Italy, including the psychiatric clinic of the University of Pisa, during the acute phase of the pandemic (24, 25).

To date there is still a lack of data on the impact of COVID-19 pandemic on psychiatric patients, particularly on those affected by BD. The aim of the present study was to examine the psychopathological impact experienced by BD patients assessed in a telepsychiatry setting displaced during the peak of the COVID-19 epidemic and the strict lockdown and social-distancing measures in a major University hospital in central Italy (Pisa). In particular, we focused on the investigation of PTSS developed in response to the COVID-19 emergency and on the possible factors associated with them.

## Methods

### Study Sample and Procedures

The present cross-sectional study included a consecutive sample of 100 subjects with a *DSM-5* diagnosis of BD enrolled at the adult outpatient psychiatric service of the Azienda Ospedaliera Universitaria Pisana (AOUP, Pisa, Italy) while followed in the framework of a telepsychiatry service, set up during the acute phase of the COVID-19 pandemic. This specific service was introduced from 1 March 2020 to carry on the psychiatric care of BD patients followed at the AOUP, during the lockdown phase of the COVID-19 emergency. Patients with a manic episode, severe depression, catatonia, active alcohol or substance abuse, or cognitive impairment were excluded from the tele-psychiatric service. Patients were contacted once a week by an expert psychiatric clinician (BB, BFM, CA, and MA) for psychiatric control visits. The study enrollment and assessment were conducted from 1 April 2020 to 30 April 2020. Patients were evaluated in the framework of the telepsychiatry visit. All eligible subjects were asked to provide written informed consent after receiving a complete description of the study and they had the opportunity to ask questions. The study was conducted in accordance with the Declaration of Helsinki and approved by the Ethics Committee of Area Vasta Nord-Ovest Toscana (Italy).

### Instruments and Assessments

All subjects were assessed by means of the Impact of Event Scale- Revised (IES-r) (26) to investigate PTSS; Generalized Anxiety Disorder 7-Item (GAD-7) (27) to explore anxiety symptoms; the Hamilton Depression Rating Scale (HAM-D) (28) to evaluate depressive symptoms; and Young Mania Rating Scale (YMRS) (29) to examine manic symptoms. We also gathered the socio-demographical and clinical data through a specific datasheet reporting information on the COVID-19 pandemic. Expert psychiatric clinicians (BB, BFM, CA, and MA) performed clinical interviews and ratings, while self-report scales were sent to patients by email, completed, and then sent back immediately after the visit.

The IES-r is a 22-item scale measuring three core features of PTSD (re-experiencing of traumatic events, avoidance, and hyperarousal) and thus items, coded on a 0–4 scale, are divided into three subscales: *intrusion, avoidance*, and *hyperarousal*. All items refer to the last week prior to the assessment. The questionnaire has an adequate internal consistency and high test-retest reliability (*r* = 0.93). The mean score of the items of each subscale determines the subscale score. The IES-r total score is calculated adding the score of each item. A score over 32 represents a cutoff for PTSS (30). In accordance with the aim of the study, the items referred to the traumatic events that the subjects had experienced in the framework of the COVID-19 pandemic.

The GAD-7 is a self-assessment questionnaire used as a tool for screening and measuring the severity of anxious symptoms. Particularly, it investigates the frequency of anxious symptoms in the last 2 weeks using seven items with a score ranging from 0 (never) to 3 (almost every day). In the validation study, the internal consistency of the scale was excellent (alpha = 0.92). Scores over 10 suggest the presence of moderate to severe anxiety symptoms.

The HAM-D is the most widely used clinician administered scale to evaluate the presence and the severity of depressive symptoms. It consists of 21 items, some of which are assessed on scales from 3 to 5 points, with well-defined severity levels. The total score is assessed on the first 17 items, and a score over 17 suggests the presence of moderate–severe depression.

The YMRS is the most widely clinician administered scale used for the assessment of the severity of manic symptoms. The scale is composed by 11 items: four items are graded on a 0–8 scale, while seven items are graded on a 0–4 scale. The score for each item is summed to obtain the YMRS total score and a score over 20 is usually considered a cut-off for a manic episode. Internal consistency of the instrument is good (alpha = 0.72).

### Statistical Analysis

Continuous variables were reported as mean ± standard deviation (SD), whereas categorical variables were reported as percentages. All tests were two-tailed and a *p*-value < 0.05 was considered statistically significant. All statistical analyses were performed using the Statistical Package for Social Science, version 25.0 (SPSS Inc.).

Chi-square test (or Fisher test if appropriate) was computed to compare socio-demographical and clinical characteristics between individuals with and without PTSS. We computed the non-parametric Mann-Whitney test for the comparisons between subjects with PTSS and those without PTSS of the not normal distributed variables GAD-7, HAM-D, and YMRS scores.

A multiple logistic regression model was utilized to study the strongest predictor of PTSS (dependent variable) among the predictors associated with PTSS in the univariate analysis. Accordingly, gender, work, or social difficulties due to the lockdown, GAD-7, HAM-D, and YMRS total scores were used as independent variables.

## Results

The sample included 36 (36%) males and 64 females (64%). The mean age was 47.04 ± 16.18 (min 19, max 81, median 48). Thirty-eight subjects (38%) were married or cohabiting, 23 (23%) lived alone, 17 (17%) had a University degree, and 48 (48%) were employed. Furthermore, 80 (80%) had a psychiatric family history, 23 (23%) reported a comorbid *DSM-5* anxiety disorder, and 6 (6%) a *DSM-5* obsessive-compulsive disorder. For what concerned the psychopharmacological treatment: 78 (78%) were on antidepressants, 88 (88%) mood stabilizers, 58 (58%) antipsychotics, and 23 (23%) benzodiazepines.

In the framework of the COVID-19 pandemic, 31 subjects (31%) reported to be at risk for medical complications in the case of the COVID-19 infection, one (1%) was positive to COVID-19, and 27 (27%) reported work and economic difficulties due to the lockdown. Moreover, 32 individuals (32%) had a close one at risk of the COVID-19 infection, 8 (8%) a relative or a close one infected by COVID-19, and 3 (3%) a loss of a relative or a close one by COVID-19. Socio-demographical and clinical characteristics of the sample are summarized in Table 1.

**Table 1:** Socio-demographical and clinical characteristics in the total sample (*N* = 100), patients with (*N* = 17) and without (*N* = 83) acute PTSS.

	**Total Sample *N* (%)**	**No-PTSS*N* (%)**	**PTSS *N* (%)**	***p***
Age > 48	50 (50%)	10 (51.2%)	7 (41.2%)	0.626
Females	64 (64%)	49 (59.0%)	15 (88.2%)	**0.045**
Married/cohabiting	38 (38%)	33 (39.8%)	5 (29.4%)	0.599
Living with family	23 (23%)	64 (77.1%)	13 (76.5%)	0.594
University degree	17 (17%)	14 (16.9%)	3 (17.6%)	1.000
Employed	48 (48%)	39 (47.0%)	9 (52.9%)	0.856
Psychiatric Family History	80 (80%)	64 (77.1%)	16 (94.1%)	0.182
Anxiety Disorder	23 (23%)	17 (20.5%)	6 (35.3%)	0.314
Obsessive Compulsive Disorder	6 (6%)	6 (7.2%)	0 (0%)	0.586
Antidepressant	78 (78%)	62 (74.7%)	16 (94.1%)	0.109
Mood Stabilizer	88 (88%)	75 (90.4%)	13 (76.5%)	0.119
Antipsychotic	58 (58%)	51 (61.4%)	7 (41.2%)	0.203
Benzodiazepine	23 (23%)	19 (22.9%)	4 (23.5%)	1.000
Being at risk for medical complications related to COVID-19 infection	31 (31%)	26 (31.3%)	5 (29.4%)	1.000
Positive to COVID-19	1 (1%)	1 (1.2%)	0 (0%)	1.000
Work or financial difficulties due to the quarantine and social-distancing measures	27 (27%)	17 (20.5%)	10 (58.8%)	**0.002**
A relative or close one at risk for medical complications related to COVID-19 infection	32 (32%)	24 (28.9%)	8 (47.1%)	0.249
A relative or close one positive for COVID-19	8 (8%)	6 (7.2%)	2 (11.8%)	0.621
Loss of a relative or close one from COVID-19	3 (3%)	2 (2.4%)	1 (5.9%)	0.432

In the total sample the IES-r total score was 18.15 ± 13.67, while the *intrusion, avoidance*, and *hyperarousal* subscales scores were 0.81 ± 0.68, 0.77 ± 0.70, and 0.91 ± 0.68, respectively. Seventeen subjects (17%) reported PTSS. Particularly, PTSS was significantly higher in females than in males [15 (23.4%) vs. 2 (5.6%), *p* = 0.045] and in patients who reported work or financial difficulties due to the lockdown with respect to those who didn't experienced it [10 (37.0%) vs. 7 (9.6%), *p* = 0.002]. The mean (±SD) GAD-7, HAM-D, and YMRS scores were 6.93 ± 4.73, 10.40 ± 6.42, and 2.58 ± 3.44, respectively. Subjects with PTSS showed significant higher GAD-7 (6.01 ± 3.99 vs. 11.41 ± 5.57, *p* < 0.001) and HAMD (9.36 ± 5.89 vs. 15.47 ± 6.66, *p* < 0.001) scores with respect to those without PTSS. Twenty-six (26%) subjects showed moderate to severe anxiety symptoms, 10 (58.8%) with PTSS and 16 (19.3%) without PTSS (*p* = 0.002). Moderate/severe depressive symptoms were reported by 17 (17%) subjects, 7 (41.2%) with PTSS and 10 (12.0%) without PTSS (*p* = 0.008). No subjects reported a manic episode (see Table 2 for details).

**Table 2:** Comparison of GAD-7, HAM-D, and YMRS total scores between subjects with (*N* = 17) and without (*N* = 83) acute PTSS.

	**Total Sample mean ± SD**	**No-PTSSmean ± SD**	**PTSS mean ± SD**	***p***
GAD-7	6.93 ± 4.73	6.01 ± 3.99	11.41 ± 5.57	** <0.001**
HAM-D	10.40 ± 6.42	9.36 ± 5.89	15.47 ± 6.66	** <0.001**
YMRS	2.58 ± 3.44	2.65 ± 3.54	2.23 ± 3.01	0.645
	*N* (%)	*N* (%)	*N* (%)	
Moderate/severe anxiety symptoms	26 (26)	16 (19.3)	10 (58.8)	**0.002**
Moderate/severe depressive symptoms	17 (17)	10 (12.0)	7 (41.2)	**0.008**
Moderate/severe manic symptoms	0 (0)	–	-	**–**

In a logistic regression model, considering gender and work and economic difficulties, besides the GAD-7, HAM-D, and YMRS scores as independent variables, and the PTSS as the dependent variables, the work and economic difficulties [*b* = 1.641 (SE = 0.757), *p* = 0.030], the GAD-7 [*b* = 0.233 (SE = 0.110), *p* = 0.034], and YMRS [*b* = −0.301 (SE = 0.143), *p* = 0.036] total scores showed a statistically significant association with the PTSS. See Table 3.

**Table 3:** Logistic regression model: Gender, work, or economic difficulties GAD-7 score, HAM-D score, and YMRS score as predictive variables associated with acute PTSS in the total sample (*N* = 100).

**Predictive factors**	**b (S.E.)**	**β**	**CI 95%**	***p***
Gender	1.037 (0.946)	2.820	0.441–18.028	0.273
Work or financial difficulties due to the quarantine and social-distancing measures	1.641 (0.757)	5.158	1.171–22.723	**0.030**
GAD-7	0.233 (0.110)	1.263	1.017–1.567	**0.034**
HAM-D	0.089 (0.070)	1.093	0.953–1.253	0.202
YMRS	−0.301 (0.143)	0.740	0.559–0.980	**0.036**
K	−5.345 (1.201)	0.005	–	**0.000**

*R^2^ = 0.279; R^2^ corrected = 0.466. Global-goodness-fit percentage = 89.0%*.

## Discussion

The present study first explored the onset of acute PTSS, anxiety, and depressive symptoms in a sample of 100 patients with BD evaluated in the framework of a telemedicine service set up in the acute phase of the COVID-19 pandemic in Italy, during the period of national lockdown and ongoing social distancing measures. Seventeen patients reported PTSS, while 26 showed moderate to severe anxiety symptoms and 17 moderate to severe depressive symptoms. Work and financial difficulties, besides anxiety symptoms, appeared to be positively associated with the development of PTSS. Interestingly, acute manic symptoms seemed to be protective.

In the present study, PTSS rates were lower than those found in the majority of previous studies on patients with BD (9, 10). This difference may be related to several factors, such as the unique nature of the traumatic event “pandemic,” the assessment of PTSS in the framework of the acute phase of the pandemic threat, or the euthymic state of the patients at the beginning of the lockdown phase. Nevertheless, these rates appear to be worth clinical attention, considering that PTSS was associated with increased clinical severity of the BD, suicidal behaviors, and worsened quality of life (9, 31, 32). Further, the continuous support and the monitoring provided to the subjects by the tele-psychiatric service might be useful in preventing or attenuating the development of such PTSS (33, 34). As we expected, female patients showed higher PTSS rates than male ones. This result is in line with recent studies on the COVID-19 emergency, reporting greater PTSD rates among females (35). It is well-recognized that female gender is associated with greater vulnerability to development of pathological reactions following traumatic or stressful events, both in the general population (36, 37) and in individuals with BD (9, 38). Interestingly, work and financial problems appear to be strictly associated with PTSS symptom severity. Several authors highlighted the relationship between unemployment or financial difficulties and poor mental health outcomes, such as depression, anxiety, or suicide behaviors (39–41). Elbogen et al. (42), in a sample of 1,388 Iraq and Afghanistan War Veterans, found that subjects with poor money incomes were significantly more likely to be affected by PTSD and to report a wide range of reckless, impulsive, or self-destructive behaviors. The impact of low economic status on the development of acute PTSS or PTSD was also reported after traumatic injury (43) or mass trauma (44). Furthermore, PTSS *per se* can affect global functioning levels, work abilities, and consequently the socioeconomic status (42, 45). In this vicious cycle, the results of the present study highlight the relevance of early detection and treatment of PTSS and PTSD, also in consideration of the possible economic implications in the long-term of the COVID-19 pandemic not yet overcome.

In regard to the other psychopathological features of the sample, we observed substantial percentages of anxiety and depressive symptoms, especially in patients reporting PTSS. High rates of depression and anxiety emerged in several different populations during the COVID-19 pandemic (4–6, 46, 47) and our data corroborated the assumption that they represented, together with PTSS, the most common psychopathological reactions to the outbreak. Furthermore, the greater depressive and anxiety symptoms burden among subjects with PTSS is so well-recognized in literature that a lively debate is ongoing on the boundaries between these disorders (37, 48). We also found low rates of manic symptoms in the sample during the acute phase of the pandemic. However, to the best of our knowledge, there is still a lack of data on this topic, so it is not easy to compare our results. Previous studies explored depressive or manic reactions related to other categories of traumatic or stressful events. However, these studies focused not on the acute phase but later on in the aftermath of a traumatic event. Particularly, some authors pointed out that in patients with BD, negative life events, such as job loss, bereavement, or personal issues, could worsen not only depressive symptoms but also manic ones (31, 49, 50). In the present study, the quarantine and social-distancing measures related to the COVID-19 pandemic showed to be less likely related to a manic-hypomanic episode, and a possible interpretation of this result could suggest an effective role of tele-psychiatry monitoring, and the consequent treatment changes, in preventing or alleviating manic manifestations.

Surprisingly, the presence of manic symptoms appeared also to be protective for the development of PTSS. This result is in contrast with existent literature on the role of manic/hypomanic state during a traumatic event as a vulnerability factor for the subsequent onset of PTSD symptoms (51–54). Nevertheless, it is worth noticing that manic symptoms levels in the present study were low, with no patient reaching the threshold for manic episode. Furthermore, the relationship between the presence of these symptoms and the development of future PTSD cannot be determined with our data and needs to be further assessed. Conversely, in the regression analysis financial difficulties and anxiety symptoms were associated with PTSS. In particular, a great amount of research reported that anxiety, fear, and distress at the time of the trauma were strictly associated with the development of post-traumatic stress reactions (55). Comorbid anxiety was related to an increased risk of PTSD onset both in general population and in longitudinal studies on BD patients (51, 52, 56). Our results corroborate these previous findings in a bipolar sample exposed to the “COVID-19 outbreak” event.

Some limitations should be kept in mind when interpreting the results of the study. The first limitation was the small sample size. However, we may argue that it represented the first homogeneous sample of subjects with BD followed in a telemedicine service during the COVID-19 pandemic. Second, we took advantage of self-report instruments to detect PTSS and anxiety that could be considered less accurate than a clinician assessment. Third, not all possible confounders and relevant COVID-19 related stressors were considered, and thus further studies may be warranted. Finally, the lack of a control group not followed in tele-psychiatry did not allow us to investigate the efficacy of this methodology beyond speculation. However, evaluation of efficacy was not an aim of the study.

In conclusion, the stress related to the acute phase COVID-19 outbreak could lead patients with a severe psychiatric disorder, such as BD, to develop psychopathological reactions such as depression, anxiety, and PTSS. Our results suggest that females who experienced work or financial difficulties may need greater attention in order to prevent possible PTSS. Further studies are needed to assess the psychopathological trajectories of patients with BD after the COVID-19 pandemic and the possible therapeutic strategies that could be useful to prevent negative outcomes in this population.

## Data Availability Statement

The data supporting the findings of the article are not publicly available, but it can be provided by the corresponding author on reasonable request.

## Ethics Statement

The studies involving human participants were reviewed and approved by Comitato Etico Regionale per la Sperimentazione Clinica della Regione Toscana AREA VASTA NORD OVEST (CEAVNO, Pisa, Italy). The patients/participants provided their written informed consent to participate in this study.

## Author Contributions

All authors gave substantial contribution to the study and approved the final version of the manuscript and the manuscript submission to Frontiers in Psychiatry.

## Conflict of Interest

The authors declare that the research was conducted in the absence of any commercial or financial relationships that could be construed as a potential conflict of interest.
